# Reduction of microhemorrhages in the spinal cord of symptomatic ALS mice after intravenous human bone marrow stem cell transplantation accompanies repair of the blood-spinal cord barrier

**DOI:** 10.18632/oncotarget.24360

**Published:** 2018-01-31

**Authors:** David J. Eve, George Steiner, Ajay Mahendrasah, Paul R. Sanberg, Crupa Kurien, Avery Thomson, Cesar V. Borlongan, Svitlana Garbuzova-Davis

**Affiliations:** ^1^ Center of Excellence for Aging and Brain Repair, University of South Florida, Morsani College of Medicine, Tampa, FL, USA; ^2^ Department of Neurosurgery and Brain Repair, University of South Florida, Morsani College of Medicine, Tampa, FL, USA; ^3^ Department of Pathology and Cell Biology, University of South Florida, Morsani College of Medicine, Tampa, FL, USA; ^4^ Department of Psychiatry, University of South Florida, Morsani College of Medicine, Tampa, FL, USA; ^5^ Department of Molecular Pharmacology and Physiology, University of South Florida, Morsani College of Medicine, Tampa, FL, USA

**Keywords:** amyotrophic lateral sclerosis, symptomatic ALS mice, microhemorrhage, human bone marrow CD34^+^ cells, blood-spinal cord barrier

## Abstract

Blood-spinal cord barrier (BSCB) alterations, including capillary rupture, have been demonstrated in animal models of amyotrophic lateral sclerosis (ALS) and ALS patients. To date, treatment to restore BSCB in ALS is underexplored. Here, we evaluated whether intravenous transplantation of human bone marrow CD34^+^ (hBM34^+^) cells into symptomatic ALS mice leads to restoration of capillary integrity in the spinal cord as determined by detection of microhemorrhages. Three different doses of hBM34^+^ cells (5 × 10^4^, 5 × 10^5^ or 1 × 10^6^) or media were intravenously injected into symptomatic G93A SOD1 mice at 13 weeks of age. Microhemorrhages were determined in the cervical and lumbar spinal cords of mice at 4 weeks post-treatment, as revealed by Perls’ Prussian blue staining for ferric iron. Numerous microhemorrhages were observed in the gray and white matter of the spinal cords in media-treated mice, with a greater number of capillary ruptures within the ventral horn of both segments. In cell-treated mice, microhemorrhage numbers in the cervical and lumbar spinal cords were inversely related to administered cell doses. In particular, the pervasive microvascular ruptures determined in the spinal cords in late symptomatic ALS mice were significantly decreased by the highest cell dose, suggestive of BSCB repair by grafted hBM34^+^ cells. The study results provide translational outcomes supporting transplantation of hBM34^+^ cells at an optimal dose as a potential therapeutic strategy for BSCB repair in ALS patients.

## INTRODUCTION

Amyotrophic lateral sclerosis (ALS) is a rapidly progressing debilitative neurodegenerative disorder characterized by motor neuron degeneration in the brain and spinal cord leading to paralysis and eventual death within 3-5 years after symptom onset [1, 2]. The majority of ALS cases (90-95%) are sporadic (SALS) with unknown cause. Approximately 5-10% of cases are genetically linked (familial cases, FALS) of which 20% have a missense mutation in the Cu/Zn superoxide dismutase 1 (*SOD1*) gene [3, 4]. Additional mutations in the transactive response DNA binding protein (*TARDBP; TDP-43)*, fused in sarcoma/translocated in liposarcoma (*FUS/TLS*), angiogenin (*ANG*), and chromosome 9 open reading frame 72 (*C90RF72*) genes have been identified in FALS cases (reviewed in [5–9]); some of these mutations were noted in SALS cases. Despite the genetic variants, SALS and FALS share clinical and pathological presentations. The treatment options for ALS are mostly supportive. The only approved drugs for ALS by the United States of America Federal Drug Administration are riluzole [10] and the recently approved Radicava (edaravone).

ALS is a multifactorial disease with numerous effectors underlying disease pathogenesis such as glutamate excitotoxicity, oxidative stress, mitochondrial dysfunction, impaired axonal transport, aberrant RNA metabolism, protein aggregations, dysfunctional autophagy, modified glial cell function, altered neurotrophic factor levels, immune reactivity, and neuroinflammation (reviewed in [11–23]). Accumulating evidence [24–31] has also shown breakdown of the blood-central nervous system-barrier (B-CNS-B), i.e. the blood-brain barrier (BBB) and the blood-spinal cord barrier (BSCB), potentially representing an additional pathogenic mechanism identifying ALS as a neurovascular disease [32]. The essential role of the B-CNS-B is to maintain homeostasis within the CNS by preventing diffusion of detrimental factors from the blood circulation to the CNS [33–35]. The barriers are composed of endothelial cells and tight junctions that interact with pericytes, astrocytes, perivascular macrophages and the basal lamina to form an integrated microvascular unit [33]. Originally, we demonstrated B-CNS-B impairment in ALS patients [25] and the G93A SOD1 mouse model of ALS [24, 26]. In the G93A mice, endothelial cell degeneration and astrocyte end-feet alterations have been observed before disease onset as well as at different stages of the disease [24, 26, 28]. Importantly, BSCB alterations were indicated in SOD1 mutant mice and rats prior to motor neuron degeneration and neuroinflammation [28, 29, 31], suggesting vascular damage as an early ALS pathological event. Moreover. compromised BSCB integrity was demonstrated by Evans blue dye extravasation into CNS parenchyma in pre-symptomatic [26] and symptomatic G93A rodents [29]. Reductions of tight junction proteins such as zonula occludens 1 (ZO-1), occludin, and claudin-5 have also been detected in the ventral horn of the lumbar spinal cord [28, 31] in G93A SOD1 mice at pre-symptomatic and symptomatic disease stages. However, decreased levels of tight junction proteins were determined in G93A SOD1 rats mainly at the symptomatic stages [29]. Studies using post-mortem human ALS tissues in several laboratories [25, 27, 28, 36] also support disease-related BSCB dysfunction by demonstrating endothelial cell degeneration, astrocyte end-feet alterations, and reduction of tight junction protein expressions. Thus, it is possible that the initiating pathological trigger for ALS is a dysfunctional B-CNS-B, allowing detrimental factors from the systemic circulation to penetrate the CNS and initiate inflammation fostering motor neuron degeneration [30, 36].

Microhemorrhages within the CNS parenchyma are indicative of capillary damage within the B-CNS-B [23, 29, 36]. These capillary ruptures may be identified through detection of ferric iron deposits derived from the blood compartment [29–31, 36, 37]. A significantly higher number of hemosiderin deposits have been observed in the ventral horn of the cervical spinal cord of ALS patients [36] and the ventral horn of the lumbar spinal cord in a rodent model of ALS [29–31]. However, the distribution of microhemorrhages within the gray and white matter of both the cervical and lumbar spinal cord of ALS patients and rodents has not previously been reported.

Since BSCB dysfunction is a potential contributor to ALS pathogenesis, restoration of BSCB integrity could be an important target in treatment development for ALS. We have recently shown that unmodified human bone marrow CD34^+^ (hBM34^+^) stem cells intravenously transplanted into symptomatic ALS mice dose-dependently differentiate into endothelial cells and engraft into the capillary walls in the cervical and lumbar spinal cords at 4 weeks post-transplantation [38]. Moreover, mice treated with the highest cell dose demonstrated improvements in behavioral disease outcomes and motor neuron survival in addition to reductions of macro- and microgliosis, maintenance of perivascular end-feet astrocytes, and reduction of capillary permeability. These study results may indicate BSCB repair processes after transplantation of hBM34^+^ cells into symptomatic ALS mice. However, it is still unclear if structural and/or functional BSCB restoration occurred via cell transplantation. Although we showed that extravasation of Evans blue dye into the spinal cord was significantly reduced in ALS mice receiving the high dose of hBM34^+^ cells [38], functional BSCB repair needed to be revealed by investigating the cell transplant effect on spinal cord capillary ruptures.

The aim of this study was to determine whether intravenous transplantation of hBM34^+^ cells at different doses into symptomatic G93A SOD1 mice leads to restoration of capillary integrity by detection of microhemorrhages. A specific focus was defining the distributions of microhemorrhages in the gray and white matter of the cervical and lumbar spinal cord enlargements in the cell-treated mice vs. media-treated ALS mice.

## RESULTS

The effect of intravenous administration of different doses of hBM34^+^ cells into symptomatic G93A SOD1 mice (13 weeks old) on capillary integrity was analyzed by detection of microhemorrhages in the cervical and lumbar spinal cords. Microhemorrhages, as free iron deposits, were observed in the cervical and lumbar spinal cords of each animal 4 weeks after cell transplantation. Quantitative capillary rupture analysis was performed in the ventral horn, dorsal horn, lateral white matter, anterior white matter, and posterior white matter regions within the cervical (C4-C6) and lumbar (L3-L5) spinal cord enlargements. Of the 31 total G93A SOD1 mice used in the study, five animals (Group 1 – one, Group 3 – two, Group 4 – two) were excluded due to premature death at 15-16 weeks of age or death from anesthetic complications during cell/media administrations.

### Microhemorrhages within the cervical spinal cords of G93A mice

**Figure 1:**
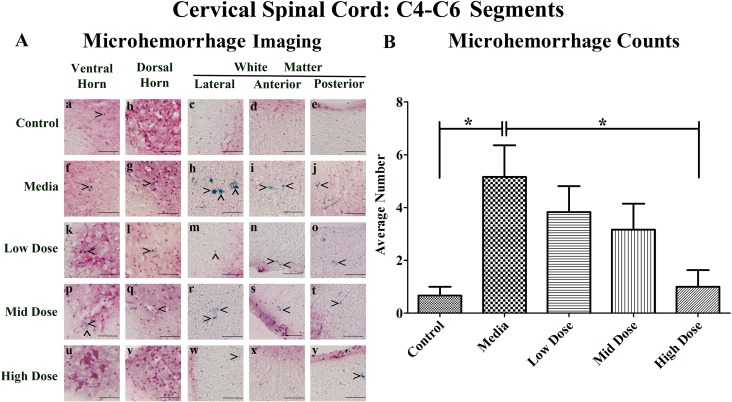
Distribution of microhemorrhages within the cervical (C4-C6) spinal cord of G93A mice after cell transplantation Tissue sections from the C4-C6 enlargement segment of the cervical spinal cord of 17 week old G93A mice were stained with Perl's Prussian blue to reveal ferric iron deposits as markers of microhemorrhages (blue). The tissue was counterstained with nuclear fast red (nuclei - red, cytoplasm - pink). **(A)** Numerous microhemorrhages (^) were detected within the parenchyma of the ventral and dorsal horns, lateral, anterior, and posterior white matter of the cervical spinal cord of ALS mice receiving media **(f-j)**, low **(k-o)**, or mid **(p-t)** cell dose. No microhemorrhages were seen in ventral or dorsal horns **(u, v)** and anterior white matter **(x)** of the high cell-dose mice. However, some small microhemorrhages were observed in the lateral white matter **(w)** and the posterior white matter **(y)** in these mice. A small iron deposit was detected in the ventral horn **(a)** of control mice. Yet, microhemorrhages were not detected in dorsal horn **(b)**, or white matter **(c-e)** in these animals. In 50% of the high cell-dose mice, no microhemorrhages were observed. Scale bar is 50 μm. **(B)** Quantitative analysis of microhemorrhage distribution in the cervical spinal cord of the control and G93A mice. Media-treated mice showed a significantly (^*^p<0.05) higher number of microhemorrhages vs. controls. Microhemorrhages decreased inversely with cell dose, reaching significance with high cell-dose vs. media-treated mice (^*^p<0.05).

Perl's Prussian blue staining revealed ferric iron deposits, consequences of microhemorrhages, within the gray and white matter parenchyma of the cervical spinal cord in all examined animals to different degrees. While microhemorrhages were rare in the control mice (Figure 1Aa-e), numerous microhemorrhages were observed in media-treated mice in all five evaluated regions (Figure 1Af-j). The microhemorrhages varied in size within each animal group with more detected in the lateral and anterior white matter of media-treated animals. Similarly to media-treated mice, ferric iron deposits were observed in analyzed spinal cord regions of the low (Figure 1Ak-o) and mid (Figure 1Ap-t) cell dose-treated mice. However, no microhemorrhages were detected within the ventral horn (Figure 1Au), dorsal horn (Figure 1Av), or anterior white matter (Figure 1Ax) of the high cell dose-treated mice. Of note, some small microhemorrhages were observed in the lateral white matter (Figure 1Aw) and the posterior white matter (Figure 1Ay) in these mice.

Quantitative analysis of microhemorrhages in the cervical spinal cords determined a significantly (p<0.05) higher number of microhemorrhages in the media-treated mice compared to controls (Figure 1B). Cell-dose-dependent decreases in the number of microhemorrhages were noted after hBM34^+^ cell transplantation compared to the media-treated mice, reductions which reached significance (p<0.05) in the high cell-dose mice. In the media-treated mice, the percentages of microhemorrhages decreased across the regions as follows: ventral horn (35.5%), dorsal horn (22.6%), anterior white matter (22.6%), lateral white matter (16.1%) and posterior white matter (3.2%) (Table 1A). A similar pattern of microhemorrhage distribution was also seen in the low and mid dose-treated mice, however, only a few iron deposits were detected in ALS mice treated with the high cell-dose. Importantly, no microhemorrhages were detected in 50% of the high cell-dose treated mice, although microhemorrhages were observed in 100% of the media, low, and mid cell-dose mice. The number of microhemorrhages within the gray matter was greater vs. the white matter in media-treated, low, and mid cell-dose treated mice, with the exception of the high cell-dose mice.

**Table 1:** Distribution of microhemorrhages in the cervical and lumbar spinal cords

Animal group	Average number of microhemorrhages ± S.E.M.	Number/percentage of microhemorrhages by spinal cord region				
		Ventral horn	Dorsal horn	Lateral white matter	Anterior white matter	Posterior white matter
**A. Cervical spinal cord**						
**Control**	0.67 ± 0.33	2/50.0	0/0.0	1/25.0	1/25.0	0/0.0
**Media**	5.17 ± 1.19	11/35.5	7/22.6	5/16.1	7/22.6	1/3.2
**Low dose**	3.83 ± 0.98	7/30.5	5/21.7	5/21.7	5/21.7	1/4.4
**Mid dose**	3.17 ± 0.98	6/31.6	5/26.3	2/10.5	4/21.1	2/10.5
**High dose**	1.00 ± 0.63	2/33.3	0/0.0	1/16.7	0/0.0	3/50.0
**B. Lumbar spinal cord**						
**Control**	0.50 ± 0.34	1/33.4	1/33.3	0/0.0	1/33.3	0/0.0
**Media**	6.50 ± 0.92	16/41.0	11/28.2	6/15.4	4/10.3	2/5.1
**Low dose**	3.67 ± 1.31	10/45.5	6/27.3	3/13.6	2/9.1	1/4.5
**Mid dose**	2.00 ± 1.06	4/33.3	3/25.0	3/25.0	2/16.7	0/0.0
**High dose**	1.17 ± 0.79	3/42.8	2/28.6	2/28.6	0/0.0	0/0.0

In the cervical (A) and lumbar (B) spinal cords of media-treated mice, higher numbers of microhemorrhages were determined in the ventral and dorsal horns compared to white matter areas. Cell-transplanted mice demonstrated a similar distribution pattern to media-treated mice with a substantial reduction of microhemorrhages in the spinal cords of ALS mice receiving the high cell-dose. No microhemorrhages were detected in cervical dorsal horn, cervical anterior white matter, lumbar anterior white matter, or lumbar posterior white matter in high cell-dose mice.

### Microhemorrhages within the lumbar spinal cords of G93A mice

**Figure 2:**
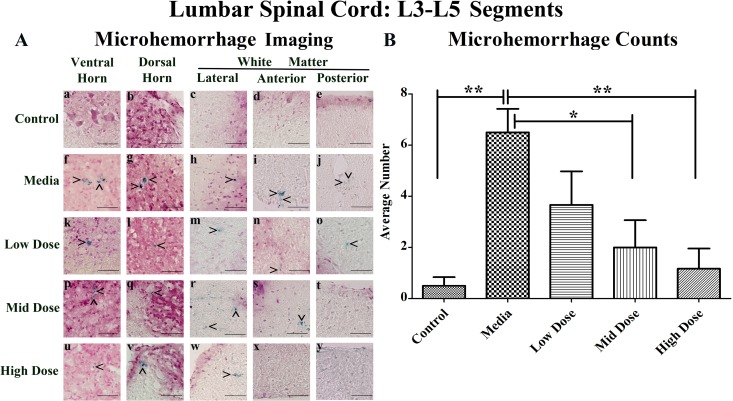
Distribution of microhemorrhages within the lumbar (L3-L5) spinal cord of G93A mice after cell transplantation Tissue sections from the L3-L5 enlargement segment of the lumbar spinal cord of 17 week old G93A mice were stained with Perl's Prussian blue to reveal ferric iron deposits as markers of microhemorrhages (blue). The tissue was counterstained with nuclear fast red (nuclei - red, cytoplasm - pink). **(A)** Distribution of microhemorrhages (^) in the gray and white matter of the lumbar spinal cord was similar to cervical spinal cord. Although no capillary ruptures were determined in gray **(a, b)** or white **(c-e)** matter of control mice, numerous microhemorrhages were observed in media-treated **(f-j)** and low cell-dose mice **(k-o)**. In mid cell-dose treated mice, iron deposits were detected in gray matter **(p, q)** and lateral/anterior white matter **(r, s)**. No microhemorrhages in posterior white matter of mid **(t)** or high **(y)** cell-dose mice or anterior white matter of high dose mice **(x)** were determined. However, some capillary ruptures were noted in gray matter **(u, v)** and lateral white matter **(w)**. No microhemorrhages were detected in 33.3% of mid cell-dose and 50% of high cell-dose mice. Scale bar is 50 μm. **(B)** Quantitative analysis of microhemorrhage distribution in the lumbar spinal cord of the control and G93A mice. Media-treated mice demonstrated a significantly (^**^p<0.01) higher number of microhemorrhages vs. controls. Microhemorrhages decreased inversely with cell dose, reaching significance with mid (^*^p<0.05) and high (^**^p<0.01) cell-doses vs. media-treated mice.

Similarly to the cervical spinal cords, microhemorrhages were rarely detected in the control mice (Figure 2Aa-e), but numerous microhemorrhages were observed throughout the lumbar spinal cord of the media-treated mice in all analyzed regions (Figure 2Af-j). The microhemorrhages varied in size within each animal group. In media-treated mice, numerous large microhemorrhages were determined mainly in the ventral horn (Figure 2Af), dorsal horn (Figure 2Ag), and anterior white matter (Figure 2Ai). Ferric iron deposits were observed in all analyzed spinal cord regions of the low cell-dose treated mice (Figure 2Ak-o). In mid cell-dose treated mice, microhemorrhages were detected in the gray matter (Figure 2Ap, q) and lateral or anterior white matter (Figure 2Ar, s) but not in the posterior white matter region (Figure 2At). However, microhemorrhages were identified in the gray matter (Figure 2Au, v) and lateral white matter (Figure 2Aw) of high cell-dose treated mice. In the anterior (Figure 2Ax) and posterior (Figure 2Ay) white matter of these treated mice no iron deposits were observed.

Significantly (p<0.01) more microhemorrhages were observed in the lumbar spinal cord of the media-treated mice compared to the controls (Figure 2B). Cell-dose dependent decreases in microhemorrhage numbers in comparison to media-treated mice were determined after hBM34^+^ cell transplantation, reaching significance in both the mid (p<0.05) and high cell-dose (p<0.01) mice.

In the media-treated mice, the percentage of microhemorrhages within each region of the lumbar spinal cord decreased as follows: ventral horn (41.0%), dorsal horn (28.2%), lateral white matter (15.4%), anterior white matter (10.3%), and posterior white matter (5.1%) (Table 1B). A similar pattern of microhemorrhage distribution was also seen in the low dose mice. No microhemorrhages were observed in the posterior white matter of the mid dose mice or the anterior and posterior white matter of the high cell-dose mice. Overall, no microhemorrhages were detected in 33% and 50% of the mid and high dose mice respectively, while microhemorrhages were observed in 100% of the media and low dose mice. The number of microhemorrhages within the gray matter was higher vs. white matter in media-treated and all cell treated mice.

### Topographic distribution of microhemorrhages within the spinal cords of G93A mice

**Figure 3:**
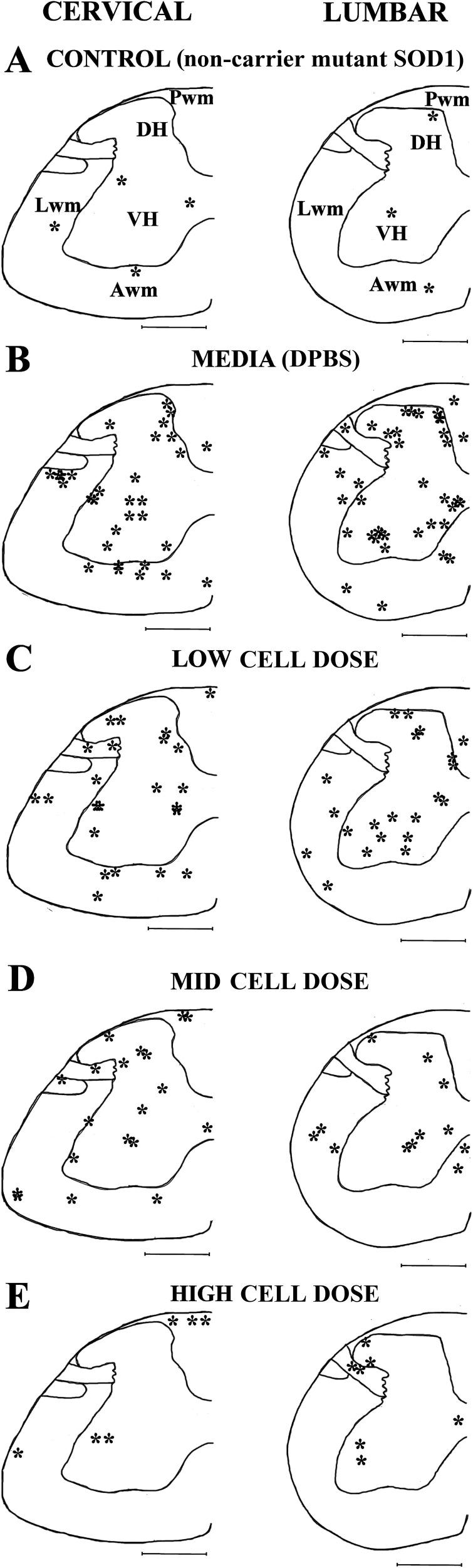
Schematic depiction of microhemorrhage distribution within the spinal cord of G93A mice after cell transplantation The locations of microhemorrhages (^*^) within the C4-C6 (left) and L3-L5 (right) segments of the spinal cord of control **(A)**, and media-treated **(B)**, low **(C)**, mid **(D)**, and high **(E)** cell-dose G93A mice are topographically mapped on the C4 and L4 segments accordingly to the mouse spinal cord atlas. In media-treated mice (B), numerous microhemorrhages were determined in the gray matter of the cervical and lumbar spinal cords. In white matter, microhemorrhages localized to ascending and descending pathways in cervical and lumbar spinal cords. Microhemorrhages decreased in gray matter of mice inversely with cell dose (C-E) with the greatest reduction observed in cervical and lumbar spinal cords of high cell-dose mice. In white matter, dose-dependent decrease of microhemorrhages (C-E) was determined only in cervical descending pathway and lumbar lateral spiny nuclei of high cell-dose mice. VH: ventral horn, DH: dorsal horn, Lwm: lateral white matter, Awm: anterior white matter, Pwm: posterior white matter. Scale bar in spinal cord section is 5mm.

Topographic distribution of microhemorrhages in the cervical and lumbar spinal cords of media-treated and cell-treated mice was analyzed by visual detection of the gray and white matter tissues accordingly to the mouse spinal cord atlas [39] and the ascending/descending pathways as described [40]. Microhemorrhage locations in the C4-C6 and L3-L5 spinal cord enlargements were mapped onto each analyzed spinal cord segment. Figure 3 presents overall microhemorrhage distribution in the gray and white matter for each animal group at C4 and L4 segments.

In the cervical spinal cords of control mice, a few microhemorrhages were detected in the gray and white matter (Figure 3A). Within the ventral horn of media-treated mice, the majority of microhemorrhages were noted in the ventral horn of lamina 7Sp and some were observed in lamina 9Sp (Figure 3B). The number of microhemorrhages decreased within these regions of the cervical spinal cord in a cell-dose dependent fashion after hBM34^+^ cell transplantation. However, numerous iron deposits were still identified in low and mid cell-dose treated mice, mainly at level of lamina 7Sp (Figure 3C, 3D). No microhemorrhages were determined at level of lamina 9Sp and only a few iron deposits were observed at lamina 7Sp in mice receiving the high cell-dose (Figure 3E).

Microhemorrhages within the cervical dorsal horn of media-treated mice were primarily localized in the 3Sp, 4Sp, and 5Sp laminas (Figure 3B). Microhemorrhage numbers decreased with escalating cell doses (Figure 3C, 3D). In high cell-dose treated mice no microhemorrhages were observed within the dorsal horn (Figure 3E). In the cervical white matter of the media-treated mice, many microhemorrhages were determined in the lateral and anterior white matter (Figure 3B) corresponding to areas of ascending and descending pathways, such as the spinothalamic and reticulospinal tracts. Fewer iron deposits were found in low and mid cell-dose treated mice (Figure 3C, 3D). No microhemorrhages were observed in the high cell-dose mice within the anterior white matter area related to the descending pathways (Figure 3E).

In contrast to the cervical spinal cord, the topographic distribution of the microhemorrhages within the ventral horn of the lumbar spinal cord in media-treated mice revealed similar locations in laminas 7Sp and 9Sp (Figure 3B). A dose-dependent decrease in microhemorrhages was evident in mice treated with low and mid cell-doses (Figure 3C, 3D). Only a few microhemorrhages were present at lamina 9Sp in the high-dose mice (Figure 3E). In the dorsal horn of the lumbar spinal cord, microhemorrhages were found throughout laminas 1Sp-6Sp in the media-treated mice (Figure 3B). The overall cell-dose dependent decrease of microhemorrhages in the lumbar spinal cord mirrored patterning of iron deposits in the cervical dorsal horn (Figure 3C-3E). Only a small number of microhemorrhages were found within laminas 2Sp and 3Sp of the high cell-dose treated mice (Figure 3E). Within the lumbar white matter of the media-treated mice, noted microhemorrhages corresponded with the locations of ascending (spinocerebellar and spinothalamic) and descending (corticospinal and reticulospinal) pathways (Figure 3B). In the lumbar white matter of the ALS mice treated with low or mid cell doses, a few iron deposits were determined, mainly within the lateral white matter corresponding to ascending pathways (Figure 3C, 3D). In high cell-dose treated mice, microhemorrhages were only observed in the area of the lateral spiny nucleus (Figure 3E).

## DISCUSSION

In the present study, the effect of intravenously administering a low, mid or high dose of hBM34^+^ cells into symptomatic G93A SOD1 mice was explored to determine the presence of microhemorrhages 4 weeks post-treatment, as an indicator of putative BSCB repair. The major study findings were: (1) widespread distribution of microhemorrhages within the gray and white matter parenchyma of the cervical and lumbar spinal cord of late symptomatic media-treated G93A mice; (2) more microhemorrhages in the gray matter vs. white matter of the spinal cords in media-treated ALS mice; (3) higher microhemorrhage occurrence in the lumbar vs. the cervical spinal cord in media-treated ALS mice; (4) significant decrease of microhemorrhages in the cervical spinal cords of high cell-dose G93A mice; and (5) significant decrease of microhemorrhages in the lumbar spinal cords of mid and high cell-dose G93A mice. Pervasive microvascular ruptures determined in the gray and white matter of the cervical and lumbar spinal cords in late symptomatic ALS mice are important findings. This vascular pathology in ALS may have implication for disease pathogenesis and highlights the microvasculature as a novel therapeutic target. Here, we are the first to demonstrate, from a translational viewpoint, that administration of hematopoietic stem cells derived from bone marrow at symptomatic disease stage prevents capillary rupture, potentially leading to BSCB repair in ALS. Although the high dose of 1 × 10^6^ cells provided the most beneficial effect on restoration of capillary integrity in the cervical and lumbar spinal cords at 4 weeks post-transplantation, the mid (1 × 10^5^) cell dose also demonstrated a significant decrease of microhemorrhages in the lumbar spinal cord, suggesting a range of therapeutic cell doses for hBM34^+^ cells to be effective in ALS. These data support our previous study results showing that transplantation of hBM34^+^ cells into symptomatic ALS mice, specifically with the higher cell-doses, reduced Evans blue extravasation into spinal cord parenchyma and delayed disease progression [38]. Thus, reduction of microhemorrhages in the spinal cords of symptomatic ALS mice via administration of mainly the higher doses of hBM34^+^ cells confirms potential vascular repair towards BSCB restoration in ALS.

Numerous reports have demonstrated structural and functional impairment of B-CNS-B in ALS patients [25, 27, 28, 36] and in animal models of disease [24, 26, 28–31] including vascular leakage or even microhemorrhages. Results of our present study showed numerous microhemorrhages within the parenchyma of the lumbar spinal cord in late symptomatic ALS mice, supporting previous findings on capillary ruptures in this spinal cord segment in a rodent model of ALS [28–31]. However, to our knowledge, our present study is the first to demonstrate microhemorrhages within the gray and white matter of the cervical spinal cord of symptomatic ALS mice. Of note, microhemorrhages have been previously reported in post-mortem cervical spinal cords from ALS patients [36]. However, no microbleeds were detected in twelve ALS patients with disease duration averaging 14.3 months using the T2-weighted whole-brain imaging by 7 Tesla magnetic resonance imaging (MRI) [41], possibly due to undetectable microhemorrhages at this early stage of the disease. A lack of detectable capillary ruptures in ALS patient's brains does not preclude microhemorrhages in the spinal cords of ALS patients [42], which likely present as subtle pathological microvessel alterations.

Although our quantitative data showed that both the cervical and lumbar spinal cords of media-treated symptomatic mice showed microhemorrhages, the lumbar spinal cord tended to have more microhemorrhages than the cervical spinal cord in these mice. This difference in media-treated mice at 17 weeks of age was primarily due to a greater number of gray matter (ventral + dorsal horns) microhemorrhages in the lumbar (total number: 27) than in the cervical (total: 18) spinal cord. Our study results demonstrating a higher incidence of microhemorrhages in the ventral horn vs. the dorsal horn in the lumbar segments of media-treated symptomatic ALS mice, potentially leading to motor neuron loss, are supported by previously published data [31] showing significantly more hemosiderin deposits in the anterior horn than in posterior horn of the lumbar spinal cord of different SOD1 mutant mice even at pre-symptomatic disease stage. Similarly, deposition of hemosiderin was observed in the ventral horn of the lumbar spinal cords around blood vessels and at close proximity of motor neurons in about 50% of G93A SOD1 rats at pre-symptomatic or symptomatic disease stages [29]. However, the authors showed that Evans blue dye extravasation was significantly increased in the brainstem and spinal cord of ALS rats only at symptomatic stage. This discrepancy between hemosiderin deposits and Evans blue capillary permeability in the spinal cords of rats modeling ALS needs more comprehensive evaluation in relation to disease progression.

Our other study finding is that the number of white matter (lateral + anterior + posterior) microhemorrhages was similar between the cervical (total number: 13) and lumbar (total number: 12) spinal cord segments in late symptomatic ALS mice. Interestingly, it has been shown that pre-symptomatic G93A mice demonstrated hemosiderin deposits in the lateral, anterior, and posterior funiculi with most detected in the lateral funiculus [31]. Unfortunately, the authors did not investigate the possibility of capillary ruptures in the cervical spinal cords of these mice.

Microhemorrhage, or capillary rupture, leads to blood-borne compounds exiting the capillary lumen into the surrounding CNS tissue parenchyma. Capillary ruptures, leading to the extravasation of erythrocytes, have been observed in various CNS disorders such as multiple sclerosis [37], cerebral ischemia [43] and post-traumatic epilepsy [44]. Extravasated red blood cells release free iron, a product of hemoglobin degradation, considered toxic to neurons, and this free iron might promote neuronal cell death through generation of free radicals [45]. The toxic effect of hemosiderin has been shown to contribute to early motor neuron dysfunction via an iron-dependent mechanism in ALS mice, indicating a loss of BSCB integrity [30]. Our study takes advantage of the fact that ferric iron can be detected by Prussian blue staining and the ferric iron-containing complex of polysaccharides and proteins from the metabolism of erythrocytes and other blood-borne components, known as hemosiderin, can be found in the surrounding parenchyma following capillary rupture. Other toxic entities also exist in the blood, including thrombin and fibrinogen (metabolized to fibrin), which can have inflammatory, angiogenic and neurotoxic effects on nearby cells by entering the CNS parenchyma following capillary rupture and contribute to neurovascular damage in mouse models of Alzheimer's disease [46, 47]. Our previous studies have shown perivascular fibrin deposits in the spinal cords of an ALS mouse model [26] and ALS patients [25], supporting the possibility of toxic moieties within the CNS parenchyma due to BSCB breakdown. Fibrin and thrombin accumulations within motor neurons have also been detected following BSCB disruption in mice with a deficiency of pericytes, an important cellular component of the BSCB [48]. Additionally, studies have demonstrated the presence of immunoglobulin G (IgG) and C3/C4 complement within the spinal cord parenchyma of ALS animal models [26, 29] and ALS patients [30, 49, 50], substances which may promote motor neuron dysfunction and cell death. Thus, capillary rupture, detected prior to disease symptoms, worsened during disease progression as we demonstrated in the current study; widespread microhemorrhages in the gray and white matter of the cervical and lumbar spinal cords were determined in late symptomatic G93A mice.

In the present study, the effect of intravenously administered hBM34^+^ cells at low (5 × 10^4^), mid (5 × 10^5^) or high (1 × 10^6^) dose into symptomatic G93A SOD1 mice on reducing capillary rupture was examined by detection of microhemorrhages in the spinal cords at 4 weeks post-transplantation. A significant decrease in the number of microhemorrhages was observed in both the cervical and lumbar spinal cords of ALS mice mainly receiving the high cell dose compared to the media-treated mice. These results were consistent with those of our previous study [38] showing a substantial decrease of Evans blue dye extravasation into spinal cord parenchyma in symptomatic ALS mice after receiving the same high dose of hBM34^+^ cells. This effect might be, as the authors noted [38], due to differentiation of transplanted cells into endothelial cells and subsequent differentiated endothelial cell engraftment within numerous spinal cord capillaries, improving BSCB integrity by likely replacement of damaged endothelial cells. However, transplanted stem cells could additionally provide endogenous repair of endothelial cells in ALS by secretion of specific angiogenic factors. Recently, Ropper et al. [51] showed that transplantation of human bone marrow mesenchymal stromal stem cells (hMSCs) embedded within a PLGA scaffold into injured rat spinal cord exert beneficial effects on cell engraftment, hindlimb locomotion, and neurogenesis. The authors also provided evidence that the scaffolded hMSCs secreted laminin α2 and α5 along capillary structures proximal to the implantation site in post-injured rat spinal cord, promoting angiogenesis. Since laminin is a major component of the basal lamina (i.e. basement membrane), this extracellular matrix protein has a vital role for the maintenance of B-CNS-B integrity. We have demonstrated [26] a decrease of laminin immunoexpression in capillaries of the cervical/lumbar spinal cords in ALS mice at early and late stage disease, indicating basement membrane disruption. Presently, we are investigating laminin presence in capillary basement membrane of the spinal cords of ALS mice treated with hBM34^+^ cells and study results will be reported in an upcoming paper.

Moreover, we reported [38] decreases of macro- and microgliosis, including enhancement of perivascular astrocyte end-feet, in the spinal cords of mainly high cell-dose treated ALS mice at 4 weeks post-transplantation, concurrent with reductions of microhemorrhages in ALS mice observed in the current study. Specifically, we noted re-establishment of astrocyte end-feet capillary coverage, potentially lessening BSCB permeability and preventing capillary rupture.

Since a single intravenously administrated hBM34^+^ cell dose into early symptomatic ALS mice showed benefit towards BSCB repair, repeated cell transplantations might better encourage ongoing reparative processes of the damaged barrier. ALS-like disease symptoms quickly progress in G93A SOD1 mice and mouse lifespan is limited to 6-7 weeks after initial symptoms in this disease model. The prevalence and severity of capillary barrier damage, predominantly by endothelial cell degeneration, in the brain and spinal cords of ALS mice significantly increases during disease progression [24, 26]. Thus, repeated cell administrations (potentially weekly) might substantially contribute to ongoing replacement or endogenous repair of damaged endothelial cells over the course of the disease. Also, repeated smaller cell doses might be a better therapeutic approach for B-CNS-B repair in ALS. This study is currently underway.

Additionally, our examination of the spinal cords in control mice revealed the presence of a few microhemorrhages in the cervical and lumbar spinal cords, predominantly in the ventral horn, lateral and anterior white matter. While microhemorrhages in the control mice were rare, our finding agrees with other reports of rare microhemorrhages detected in the lumbar spinal cords of control mice or cervical spinal cords of non-neurodegenerative disease controls [30, 31, 36]. Potentially, uncontrolled fluid pressure during animal perfusion might be the cause of capillary rupture in these animals. In our study, all mice were perfused transcardially under pressure controlled delivery of phosphate buffer (PB) at 80-85mm Hg, while the normal arterial diastolic blood pressure of a mouse is approximately 102 mm Hg [52]. This technique avoids perfusion-related capillary rupture and has proven effective in our studies [24, 26, 38]. Alternatively, since angiogenesis involves the proliferation and migration of endothelial cells from already developed vessels for the sprouting of new vessels [53], newly formed vessels could have compromised integrity leading to capillary leakage. This possibility may also be true in animal models of ALS, due to continuous renewal of the endothelial cell layer within the capillary lumen. The presence of multiple layers of endothelial cells has been detected within capillaries of the brain and spinal cord in ALS mice, but not in controls, suggesting that a continuous renewal process may be responsible for replacement of damaged endothelial cells in ALS [24]. Interestingly, we showed a significant increase of microvascular density in the ventral lumbar spinal cord in sporadic ALS patients, a potential sign of compensatory neovascularization in regards to damaged capillaries in areas of motor neuron degeneration [25]. The new vessels may only partially support functional B-CNS-B properties, showing capillary leakage. Ongoing angiogenesis and increased vascular density has been observed in post-mortem tissue from patients with Alzheimer's disease [54], Parkinson's disease and progressive supranuclear palsy [55], suggesting that microvascular turnover may contribute to the occurrence of B-CNS-B dysfunction in these disorders. However, additional investigations are needed to confirm neovascularization in ALS.

Finally, our study results demonstrated widespread microhemorrhages in the spinal cords of late symptomatic ALS mice consistent with impairment of the motor neuron pathways at several levels. Microhemorrhages detected within the 9Sp lamina could directly affect cell bodies of motor neurons, while capillary ruptures within 7Sp raise the possibility of impaired interneuron communications between the dorsal sensory neurons and the ventral motor neurons. Microhemorrhages within lamina 1Sp-6Sp could degrade sensory input. Microhemorrhages within the white matter of the spinal cord could result in the toxic entities, which might interact with the myelin of the axons comprising the ascending and descending spinal cord pathways. Changes in myelin composition as well as myelin disorganization at the electron microscopy level have been reported in both pre-symptomatic and symptomatic G93A rats [56]. Also, degeneration of oligodendrocytes in the gray and white matter of the spinal cord has also been observed in both ALS patients and animal models [57, 58]. Widespread degeneration of the white matter tracts in G93A mice and ALS patients has also been reported [59–63]. Our recently published study [38] demonstrated severe astrogliosis in the reticulospinal and spinothalamic tracts of symptomatic G93A mice. However, some limitations of our current study relate to determination of lifespan and BBB repair in the brains of post-transplanted ALS mice.

In summary, pervasive microvascular ruptures determined in the gray and white matter of the cervical and lumbar spinal cords in late symptomatic ALS mice may have implications for disease pathogenesis and identify the microvasculature as a novel therapeutic target. The intravenous administration of hBM34^+^ cells into symptomatic ALS mice showed dose-dependent reductions of microhemorrhages in the cervical and lumbar spinal cords. These results support efficacy of hBM34^+^ cell transplantation at optimal cell dose as a potential future therapeutic strategy for repair of the BSCB in ALS.

## MATERIALS AND METHODS

### Ethics statement

Investigation has been conducted in accordance with the ethical standards and according to the Declaration of Helsinki and according to national and international guidelines and has been approved by the Institutional Animal Care and Use Committee at USF and conducted in compliance with the NIH's *Guide for the Care and Use of Laboratory Animals*.

The mice were maintained on a 12:12 hr. dark:light cycle commencing at 6 PM and were housed in a temperature-controlled room at 23°C. Mice had access to food and water *ad libitum*. Thirty-one transgenic male B6SJL-Tg (SOD1^*^G93A)1Gur/J mice, over-expressing human SOD1 carrying the Gly93→Ala mutation (G93A SOD1), and six non-carrier mutant SOD1 gene mice from the background strain (controls) at 7 weeks of age were received from Jackson Laboratories (Bar Harbor, ME, USA). At approximately 13 weeks of age, when initial disease symptoms such as hindlimb tremor and reduction in body weight appeared, the G93A mice were randomly assigned to one of four groups receiving hBM34^+^ cells or media: Group 1 - hBM34^+^ (5 × 10^4^ cells/mouse, low dose, n=7), Group 2 - hBM34^+^ (5 × 10^5^ cells/mouse, mid dose, n=7), Group 3 - hBM34^+^ (1 × 10^6^ cells/mouse, n=9), and Group 4- media (Dulbecco's Phosphate buffered saline [DPBS], n=8). The control mouse group (Group 5, n=6) did not receive either cell transplant or media injection.

### Cell preparation

Cryopreserved hBM34^+^ cells (All Cells, Alameda, CA, USA) were thawed rapidly at 37°C and transferred into a centrifuge tube containing 10 ml of DPBS (pH 7.4; Mediatech, Inc., Manassas, VA, USA). The cells were centrifuged at 200 × g for 10 minutes (room temperature) and the supernatant discarded. Cell viability was determined using the 0.4% trypan blue dye (Sigma-Aldrich, St. Louis, MO, USA) exclusion method. The cell concentrations were then adjusted with DPBS to obtain the required cell doses at 200μl/animal.

### Cell transplant

The G93A mice in Groups 1-3 received 5 × 10^4^, 5 × 10^5^ or 1 × 10^6^ hBM34^+^ cells in 200 μl of DPBS, respectively, via the jugular vein over a 3-5 minute period under isoflurane anesthesia (2-5% at 2L O_2_/min) as we previously described [38, 64]. The media-treated mice in Group 4 received 200 μl of DPBS. Animals in Groups 1-4 received 10 mg/kg (ip) cyclosporine A (Novartis, New York, NY, USA) daily from the time of transplant until sacrifice.

### Perfusion and tissue preparation

All cell-treated, media-treated, and control mice were sacrificed at 17 weeks of age (4 weeks after initial treatment at symptomatic disease stage) for microhemorrhage analyses in the cervical and lumbar spinal cords. Mice were sacrificed using Euthasol^®^ (0.22 ml/kg body weight) and perfused transcardially with 0.1 M PB (pH 7.2) followed by 4% paraformaldehyde (PFA) in PB solution under pressure controlled fluid delivery at 80-85 mm Hg to avoid capillary rupture. The spinal cord was then carefully removed and post-fixed in 4% PFA.

The cervical and lumbar spinal cord segments were dissected and then cryoprotected in 20% sucrose in 0.1 M PB overnight. Thirty micron coronal sections were cut on a cryostat and thaw-mounted onto slides at 150 micron intervals within the cervical and lumbar cords (i.e. every 5^th^ section). Slides were stored at −20°C until used for staining of microhemorrhages.

### Microhemorrhage staining and analysis

Perls’ Prussian blue staining was performed on the cervical and lumbar spinal cords from each animal to identify the presence of ferric iron (Fe^3+^) within the parenchyma as an indicator of microhemorrhages. The slides were thawed and hydrated in distilled water for 2 minutes, followed by transfer to 1:1 solution of 10% potassium ferrocyanide (Sigma-Aldrich) and 20% HCl for 20 minutes. After rinsing with distilled water, the slides were counterstained by nuclear-fast red (Sigma-Aldrich) solution for 5 minutes. Slides were then washed in distilled water for 5 minutes and dehydrated in increasing concentrations of ethanol (70%, 80%, 90%, 95%, and 100%) followed by xylene (2 × 3 minutes), and afterwards coverslipped with Permount^TM^ (Sigma-Aldrich).

Microhemorrhages were observed throughout the cervical and lumbar spinal cord parenchyma using an Olympus BX40 microscope with a SPOT RT3 digital camera (Diagnostic Instruments Inc., Stirling Heights, MI, USA) under bright field illumination at 20X magnification. Both the left and right sides of every 5^th^ spinal cord section (150 μm apart) were examined. The number and location of microhemorrhages within the cervical and lumbar spinal cord enlargements [38, 65] were recorded. The cervical enlargement including C4-C6 segments (14-20 sections/mouse/group) and the lumbar enlargement including L3-L5 segments (10-18 sections/mouse/group) were examined. Within these segmental regions, the gray matter was distinguished from the white matter by the nuclear-fast red counterstain and further defined as the dorsal or ventral horn based on location above or below, respectively, a line perpendicular to the midline passing through the central canal. In the cervical enlargement, the white matter was characterized as anterior (0-0.3 mm from anterior section edge, 0-1.0 mm from midline), posterior (0-0.6 mm from posterior section edge, 0-0.3 mm from midline), or lateral (0-0.4 mm from lateral section edge, 0.2-1.1 mm from posterior section edge). The white matter lumbar enlargement was characterized as follows: anterior (0-0.3 mm from anterior section edge, 0-1.0 mm from midline), posterior (0-0.6 mm from posterior section edge, 0-0.2 mm from midline), and lateral (0-0.3 mm from lateral section edge, 0.3-1.2 mm from posterior section edge). The microhemorrhages were topographically mapped for each mouse accordingly to the mouse spinal cord atlas in Watson et al [53]. Additionally, white matter microhemorrhages were further defined by the ascending and descending spinal cord pathways outlined in Watson & Harrison [52].

### Statistical analysis

Data are presented as mean ± S.E.M. and were analyzed by one way ANOVA with post hoc Tukey's Multiple Comparisons test (GraphPad Prism 5, La Jolla, CA, USA). Significance was achieved at *p*<0.05.

## Abbreviations

ALS: amyotrophic lateral sclerosis
BBB: blood-brain barrier
B-CNS-B: blood-central nervous system-barrier
BSCB: blood-spinal cord barrier
CNS: central nervous system
DNA: deoxyribonucleic acid
FALS: familial ALS
hBM34+ cells: human bone marrow cells positive for CD34 antigen
PB: phosphate buffer
PFA: paraformaldehyde
SALS: sporadic ALS
SOD1: superoxide dismutase 1
